# Expression Profiles of Dopamine-Related Genes and miRNAs Regulating Their Expression in Breast Cancer

**DOI:** 10.3390/ijms25126546

**Published:** 2024-06-14

**Authors:** Tomasz Sirek, Agata Sirek, Przemysław Borawski, Izabella Ryguła, Katarzyna Król-Jatręga, Marcin Opławski, Dariusz Boroń, Michał Chalcarz, Piotr Ossowski, Konrad Dziobek, Nikola Zmarzły, Kacper Boroń, Patrycja Mickiewicz, Beniamin Oskar Grabarek

**Affiliations:** 1 Department of Plastic Surgery, Faculty of Medicine, Academia of Silesia, 40-555 Katowice, Poland; 2 Department of Plastic and Reconstructive Surgery, Hospital for Minimally Invasive and Reconstructive Surgery in Bielsko-Biała, 43-316 Bielsko-Biala, Poland; agatasirek85@gmail.com (A.S.); katarzynakroljatrenga@gmail.com (K.K.-J.); 3 Department of Medical and Health Sciences, Collegium Medicum, WSB University, 41-300 Dabrowa Górnicza, Poland; izabella.ryg@gmail.com (I.R.); dariusz@boron.pl (D.B.); drpiotrossowski@gmail.com (P.O.); konraddziobek28@gmail.com (K.D.); nikola.zmarzly@gmail.com (N.Z.); q375@icloud.com (K.B.); pmickiewicz@wsb.edu.pl (P.M.); bgrabarek7@gmail.com (B.O.G.); 4 Independent Researcher, 87-800 Włocławek, Poland; przembor3@gmail.com; 5 Department of Gynecology and Obstetrics with Gynecologic Oncology, Ludwik Rydygier Memorial Specialized Hospital, 31-826 Kraków, Poland; marcin.oplawski@gmail.com; 6 Department of Gynecology and Obstetrics, Faculty of Medicine and Health Sciences, Andrzej Frycz Modrzewski University in Kraków, 30-705 Kraków, Poland; 7 Institute of Clinical Science, Skłodowska-Curie Medical University, 00-136 Warszawa, Poland; 8 Department of Gynecology and Obstetrics, TOMMED Specjalisci od Zdrowia, 40-662 Katowice, Poland; 9 Chalcarz Clinic-Aesthetic Surgery, Aesthetic Medicine, 60-001 Poznan, Poland; chalcarzmichal@gmail.com; 10 Bieńkowski Medical Center-Plastic Surgery, 85-020 Bydgoszcz, Poland; 11 Department of Molecular, Biology Gyncentrum Fertility Clinic, 40-055 Katowice, Poland

**Keywords:** breast cancer, microRNA, dopamine, dopaminergic system

## Abstract

This study aimed to assess the expression profile of messenger RNA (mRNA) and microRNA (miRNA) related to the dopaminergic system in five types of breast cancer in Polish women. Patients with five breast cancer subtypes were included in the study: luminal A (*n* = 130), luminal B (*n* = 196, including HER2−, *n* = 100; HER2+, *n* = 96), HER2+ (*n* = 36), and TNBC (*n* = 43); they underwent surgery, during which tumor tissue was removed along with a margin of healthy tissue (control material). The molecular analysis included a microarray profile of mRNAs and miRNAs associated with the dopaminergic system, a real-time polymerase chain reaction preceded by reverse transcription for selected genes, and determinations of their concentration using enzyme-linked immunosorbent assay (ELISA). The conducted statistical analysis showed that five mRNAs statistically significantly differentiated breast cancer sections regardless of subtype compared to control samples; these were dopamine receptor 2 (*DRD2*), dopamine receptor 3 (*DRD3*), dopamine receptor 25 (*DRD5*), transforming growth factor beta 2 (*TGF-β-2*), and caveolin 2 (*CAV2*). The predicted analysis showed that hsa-miR-141-3p can regulate the expression of *DRD2* and *TGF-β-2*, whereas hsa-miR-4441 is potentially engaged in the expression regulation of *DRD3* and *DRD5*. In addition, the expression pattern of *DRD5* mRNA can also be regulated by has-miR-16-5p. The overexpression of DRD2 and DRD3, with concomitant silencing of DRD5 expression, confirms the presence of dopaminergic abnormalities in breast cancer patients. Moreover, these abnormalities may be the result of miR-141-3P, miR-16-5p, and miR-4441 activity, regulating proliferation or metastasis.

## 1. Introduction

Breast cancer remains the most frequently diagnosed malignancy among women worldwide, as highlighted by data from the World Health Organization (WHO) in 2020. It comprised a staggering 24.5% of all cancer diagnoses and contributed to 15.5% of cancer-related deaths in women [1]. In Poland, the impact of malignant tumors is notably significant, ranking as the second leading cause of mortality. Particularly concerning is its status as the primary cause of death among women under the age of 65 over consecutive years [2]. This trend is alarming, with breast cancer alone contributing to 28.3% of deaths among young individuals aged 20 to 44 and 41.6% of deaths among middle-aged women aged 45 to 65 [2]. While cancers of the breast, lung, and large intestine are prevalent among women [3,4], breast cancer stands out as the predominant concern for both morbidity and mortality, especially among young women. According to the National Cancer Registry’s 2020 data, breast cancer accounts for a substantial 29% of both cancer incidence and mortality rates in this demographic [2]. This underscores the urgent need for targeted research and interventions to address the unique challenges posed by breast cancer, particularly among younger women, to improve outcomes and reduce the burden of this devastating disease.

Breast cancer encompasses a diverse array of diseases distinguished by variations in molecular profiles and clinicopathological features. Key disparities in estrogen receptor (ER), progesterone receptor (PgR), human epidermal growth factor receptor 2 (HER2), and the Ki67 proliferation index enable the classification of breast cancer into four primary subtypes: luminal A, luminal B, HER2-positive, and triple-negative breast cancer (TNBC) [5,6,7]. Each subtype carries distinct prognostic implications, necessitating tailored therapeutic strategies.

The luminal A subtype, characterized by the presence of estrogen and progesterone receptors and a low Ki67 proliferation index (<14% according to St. Gallen criteria), represents the most prevalent form. Exhibiting modest proliferation rates, typically lower malignancy grades, and favorable prognoses, luminal A breast cancer primarily responds to hormone therapy [8].

The luminal B subtype is divided into two cohorts, both expressing estrogen receptors. The HER2-negative luminal B subtype presents with low HER2 expression, elevated Ki67 proliferation indices (>14%), and diminished PgR expression relative to luminal A. In contrast, the luminal B HER2-positive subtype demonstrates heightened HER2 expression independent of Ki67 and PgR indices. While the prognosis is slightly inferior compared to luminal A, treatment entails a combination of hormone therapy and anti-HER2 agents for the HER2-positive subtype [9,10].

The HER2-positive (non-luminal) subtype is characterized by overexpression of the HER2 protein, driving accelerated cancer cell growth and division, alongside the absence of ER and PgR expression. This aggressive variant necessitates therapies targeting the HER2 protein [11].

Conversely, the triple-negative subtype lacks the expression of ER and PgR and lacks HER2 overexpression. TNBC is notably aggressive, with poorer prognoses compared to other subtypes, mandating chemotherapy and molecularly targeted interventions as primary treatment modalities [12].

Dopamine is a member of the catecholamine family, and in breast physiology, it has been implicated in the regulation of lactation and mammary gland development. Dopamine receptors are expressed in mammary epithelial cells, where they modulate the secretion of prolactin, a hormone crucial for milk production. Additionally, dopamine has been shown to influence cell proliferation, differentiation, and apoptosis in the mammary gland, suggesting a broader role in mammary tissue homeostasis [13,14,15,16].

The activity of dopamine is contingent upon its binding to one of its five receptors, designated as D1 through D5. These dopamine receptors (DRs) can be categorized into two groups based on their effect on adenylyl cyclase: activating receptors (DRD1 and DRD5) and inhibiting receptors (DRD2, DRD3, and DRD4) [17,18]. Furthermore, the receptors exhibit variations in binding affinity within their respective groups. For instance, DRD5 demonstrates a binding affinity ten times higher than that of DRD1, whereas DRD3 and DRD4 exhibit similar binding affinities to dopamine, albeit stronger than that of DRD2 [17,18].

Emerging evidence suggests that the dopaminergic system may play a multifaceted role in breast cancer development and progression. Dysregulation of dopaminergic signaling has been implicated in various aspects of breast cancer biology, including cell proliferation, apoptosis, migration, invasion, and angiogenesis [19,20].

Several studies have reported alterations in the expression of dopamine receptors in breast cancer tissues compared to normal breast tissue. For instance, increased expression of dopamine D2 receptors has been observed in breast cancer cells, and higher levels of D2 receptor expression have been associated with more aggressive tumor phenotypes and poorer prognosis [21,22].

Furthermore, the interplay between dopamine signaling and other pathways implicated in breast cancer pathogenesis, such as estrogen and HER2 signaling, has been elucidated. Dopamine receptors have been shown to crosstalk with estrogen receptors and HER2 receptors, modulating their downstream signaling pathways and influencing tumor growth and progression [23].

Overall, the emerging evidence linking the dopaminergic system to breast cancer highlights the complexity of tumor biology and underscores the potential significance of targeting dopaminergic signaling pathways for the development of novel therapeutic strategies in breast cancer treatment [24,25,26].

MicroRNAs (miRNAs) have emerged as critical regulators of gene expression and have garnered significant attention in the context of breast cancer research. These small non-coding RNA molecules, typically 19–22 nucleotides in length, play pivotal roles in various cellular processes including proliferation, differentiation, apoptosis, and metastasis [27,28].

In breast cancer, dysregulation of miRNAs has been implicated in the initiation, progression, and metastasis of the disease. Numerous studies have identified aberrant expression patterns of specific miRNAs in breast cancer tissues compared to normal breast tissue, highlighting their potential as diagnostic and prognostic biomarkers [29,30].

Moreover, miRNAs have been shown to influence key pathways involved in breast cancer pathogenesis, including those related to hormone receptor status, HER2/neu overexpression, and resistance to chemotherapy and targeted therapies [31]. For instance, certain miRNAs have been found to modulate the expression of estrogen receptor (ER), progesterone receptor (PR), and HER2, thereby impacting tumor growth and response to treatment [32,33].

Therefore, this study aimed to assess the expression profile of mRNA and miRNA related to the dopaminergic system in five types of breast cancer in Polish women.

## 2. Results

### 2.1. Microarray and qRT-PCR Profile of Dopamine-Related Gene Breast Cancer Samples in Comparison with Control Tissue

Out of the 175 dopamine-related mRNAs, a one-way ANOVA showed that 12 mRNAs were significantly changed in study samples in comparison to control samples (−3.0 < FC > 3.0; *p* < 0.05). Subsequently, a Tukey post hoc analysis identified the mRNAs that distinctly distinguished between breast cancer subtypes and controls, along with identifying mRNAs shared across multiple breast cancer subtypes.

**Figure 1:**
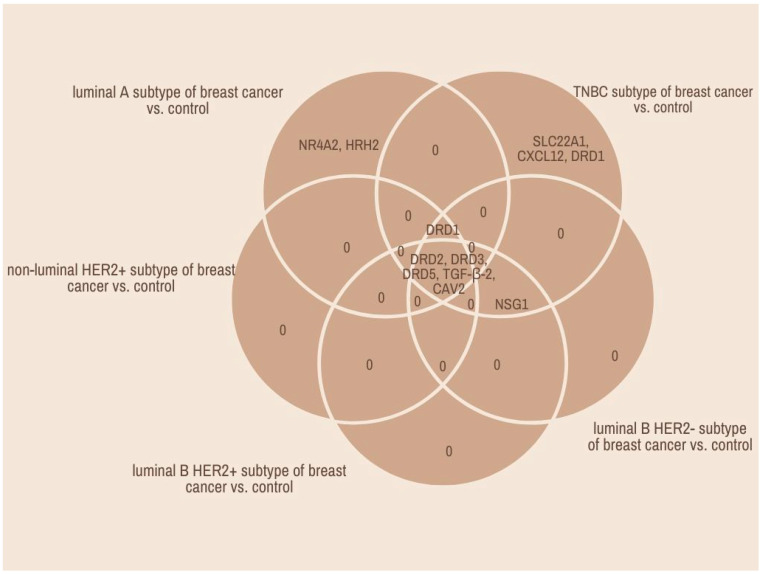
Venn diagram. *DRD2*, dopamine receptor 2; *DRD3*, dopamine receptor 3; *DRD5*, dopamine receptor 5; *TGF-β-2*, transforming growth factor beta 2; *CAV2*, caveolin 2; *SLC22A1*, solute carrier family 22 (organic cation transporter), member 1; *CXCL1*2, chemokine (C-X-C motif) ligand 12; *DRD1*, dopamine receptor 1; *NR4A2*, nuclear receptor subfamily 4, group A, member 2; *HRH2*, histamine receptor H2; *NSG1*, neuron-specific gene family member 1.

A Venn diagram revealed the genes characteristic of a given breast cancer type or common to several groups (Figure 1). Changes in the transcriptional activity of these 12 mRNAs in five subtypes of breast cancer are presented in Table 1.

The conducted statistical analysis showed that five mRNAs statistically significantly differentiated breast cancer sections regardless of subtype compared to control samples. These were *DRD2*, *DRD3*, *DRD5*, *TGF-β-2*, and *CAV2.* In contrast, changes in the expression pattern of *SLC22A1*, *CXCL1*2, and *DRD1* mRNAs were characteristic for the TNBC subtype of breast cancer, whereas *NR4A2* and *HRH2* mRNAs were characteristic for the luminal A subtype of breast cancer. In addition, *NSG1* mRNA was common for the TNBC subtype and luminal B HER2− subtype (Figure 1).

**Figure 2:**
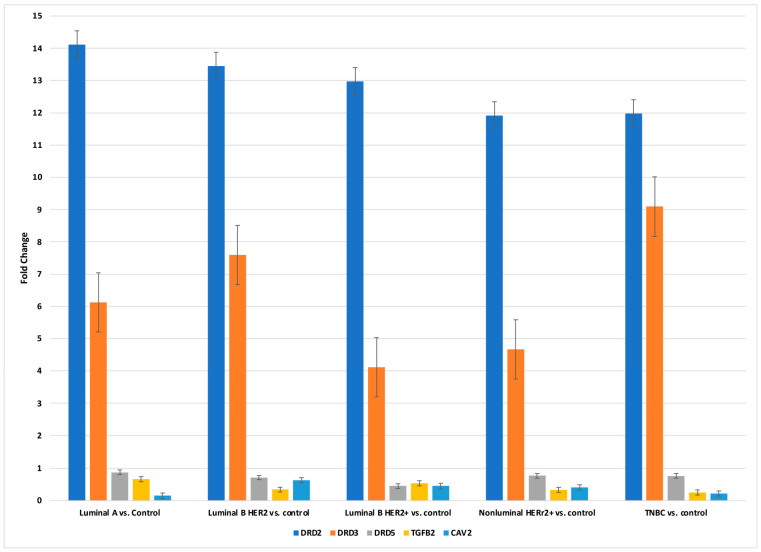
Changes in the expression profile of mRNAs in different breast cancer subtype samples compared to control samples obtained via RT-PCR. *DRD2*, dopamine receptor 2; *DRD3*, dopamine receptor 3; *DRD5*, dopamine receptor 5; *TGF-β-2*, transforming growth factor beta 2; *CAV2*, caveolin 2. Data are presented as mean ± standard deviation.

Then, to validate the microarray results, the expression profiles of *DRD2, DRD3, DRD5, TGF-β-2*, and *CAV2* were determined in breast cancer tissue samples by qRT-PCR. Figure 2 summarizes the results of this analysis (*p* < 0.05).

### 2.2. Prediction of Dopamine-Related Gene Expression Regulation by miRNAs

We further determined whether the expression of genes that differentiate breast cancer samples, regardless of type, could be regulated by miRNA molecules. The predicted analysis showed that hsa-miR-141-3p can regulate the expression of *DRD2* and *TGF-β-2,* whereas hsa-miR-4441 is potentially engaged in the expression regulation of DRD3 and DRD5. In addition, the expression pattern of *DRD5* mRNA can also be regulated by has-miR-16-5p. In contrast, the predictive analysis did not show that CAV2 expression is regulated by miRNAs in breast cancer (Table 2; *p* < 0.05).

### 2.3. Concentration of DRD2, DRD3, DRD5, TGF-β-2, and CAV2 in Breast Cancer Tissues and Control at the Protein Level

The final step of our analysis involved determining the concentration of selected proteins in the neoplastic and control tissue. The analysis indicated that the concentrations of DRD2 and DRD3 were significantly higher in breast cancer sections compared to the margin of healthy tissue (Table 3; *p* < 0.05). In contrast, the DRD5, CAV2, and TGF-β-2 concentrations in tumor tissue were significantly lower than those in control samples (Table 3; *p* < 0.05).

### 2.4. Overall Survive Analysis

**Figure 3:**
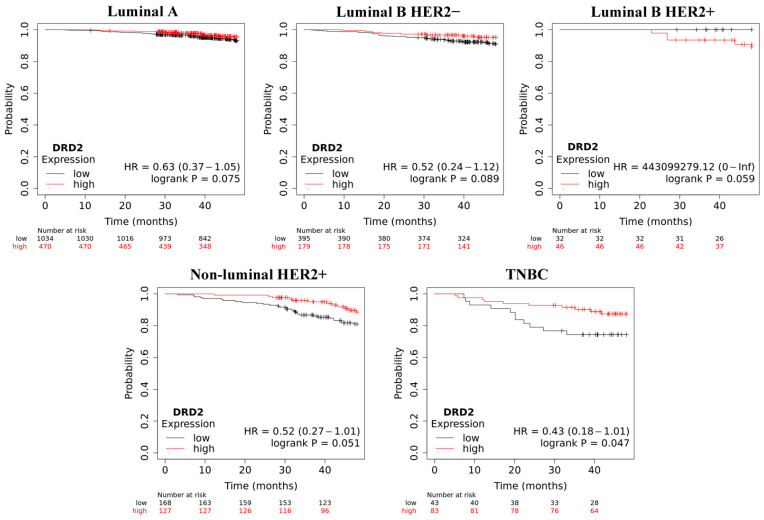
Overall survival analysis for *DRD2* in every group.

**Figure 4:**
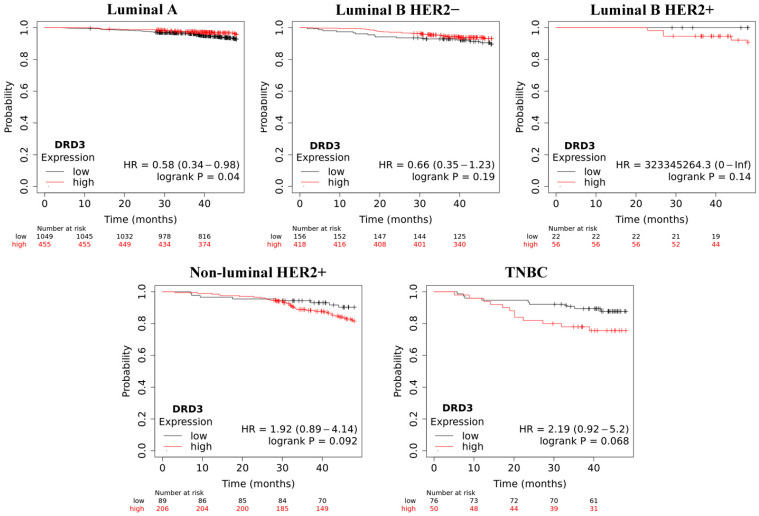
Overall survival analysis for *DRD3* in every group.

**Figure 5:**
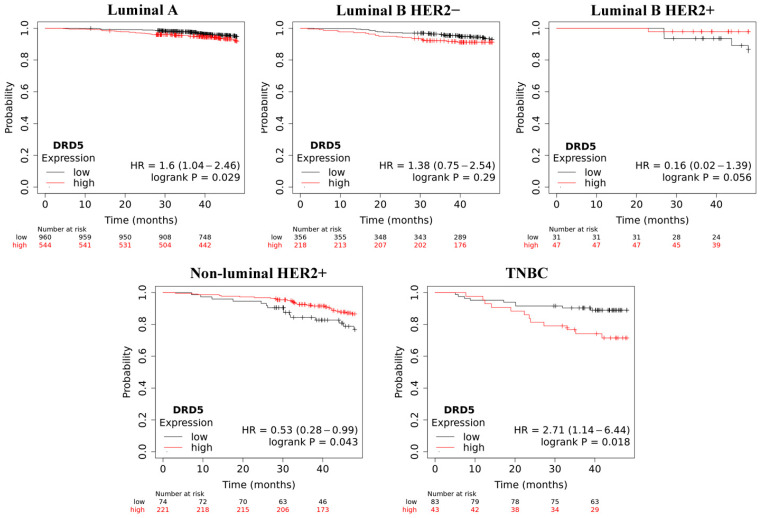
Overall survival analysis for *DRD5* in every group.

**Figure 6:**
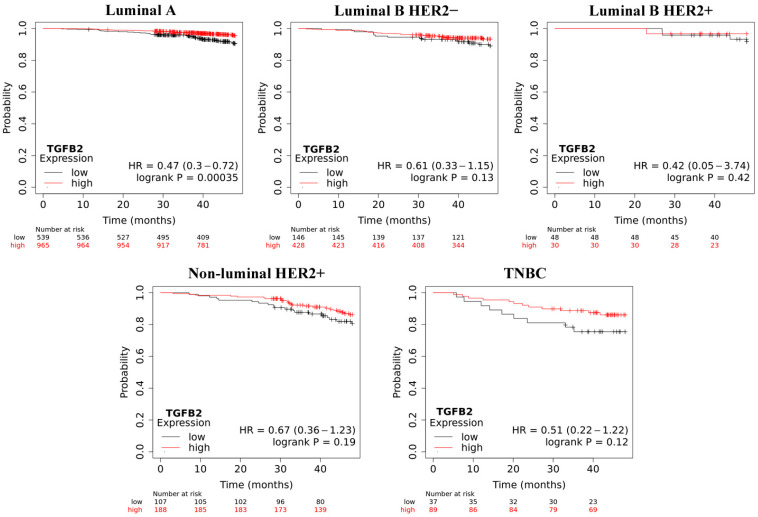
Overall survival analysis for *TGFB2* in every group.

**Figure 7:**
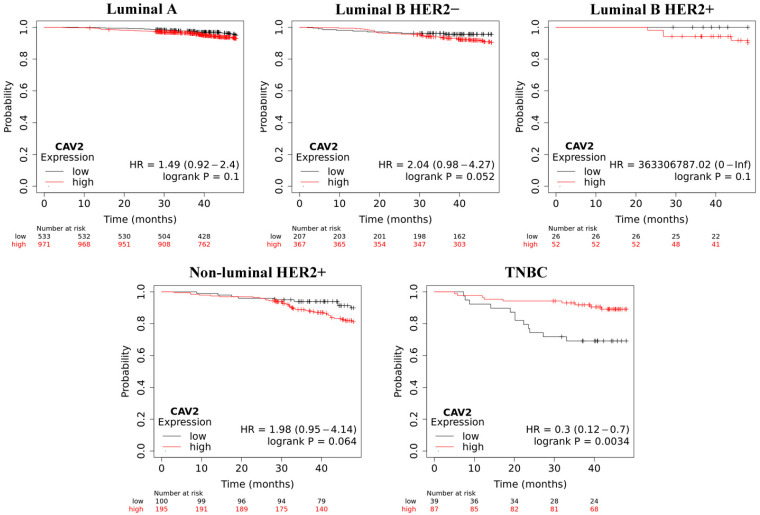
Overall survival analysis for *CAV2* in every group.

Overall survival (OS) analysis using the Kaplan–Meier plotter was performed for the studied mRNAs. The results are shown in Figure 3, Figure 4, Figure 5, Figure 6 and Figure 7.

The analysis determined that a low level of *DRD2* may promote shorter overall survival in all types of breast cancer tested, except luminal B HER2+; however, this was only statistically significant for TNBC.

In the case of *DRD3*, an inverse relationship was observed compared to *DRD2*. The largest differences were also recorded for TNBC. A significant result was recorded only for samples classified as luminal A.

Low *DRD5* levels may promote worse overall survival in luminal HER2+ and non-luminal HER2+ samples. This trend was also observed for TNBC, but it was reversed after about a year. With the exception of both luminal B groups, the observed trends were statistically significant.

Our analysis indicated that low *TGFB2* levels decreased overall survival in all samples, and this was the most pronounced in TNBC. However, this was only significant in the luminal A group. Interestingly, in our study, the activity of this gene was significantly reduced regardless of the group, which was confirmed by a low or undetectable protein level.

In TNBC samples, low CAV2 levels negatively impacted overall survival. The opposite was observed for other types of breast cancer, but it was more noticeable from around the 30th month.

### 2.5. Relationship Network for the Selected Dopamine Pathway Differentiation Genes

The study delved into the intricate relationships and characteristics of the interactions among gene products involved in the dopamine pathway using the STRING database (String Database 11.0). Within this database, the proteins encoded by the analyzed genes form a tightly interwoven network of potential protein–protein interactions, characterized by 8 edges and 10 nodes (*p* < 0.001; average local clustering coefficient 0.45, average node degree 1.60). These edges symbolize connections between proteins, with the strength of an edge indicating the likelihood of interaction between them. Importantly, these interactions denote specific and significant protein–protein associations, suggesting cooperative roles in shared functions. It is noteworthy that these interactions do not necessarily imply physical binding between the proteins.

**Figure 8:**
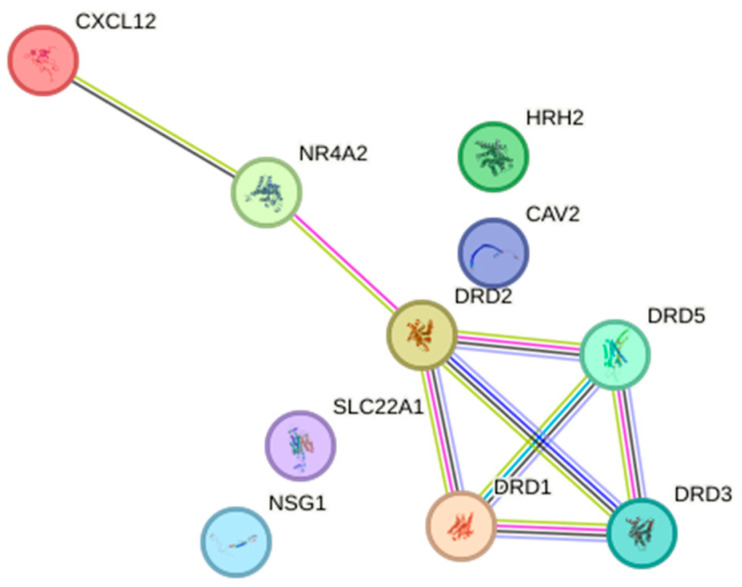
Relationship network for the selected dopamine pathway differentiation genes generated in the STRING database.

The outcomes are visually represented in a diagram showcasing the interactions between proteins (Figure 8). Additionally, the analysis classified the genes encoded by the selected participants into five biological processes, five molecular functions, and five KEGG pathways.

DRD2, dopamine receptor 2; DRD3, dopamine receptor 3; DRD5, dopamine receptor 5; TGF-β-2, transforming growth factor beta 2; CAV2, caveolin 2; SLC22A1, solute carrier family 22 (organic cation transporter), member 1; CXCL12, chemokine (C-X-C motif) ligand 12; DRD1, dopamine receptor 1; NR4A2, nuclear receptor subfamily 4, group A, member 2; HRH2, histamine receptor H2; NSG1, neuron-specific gene family member 1. The green line for interaction indicates “textmining”; the black line “co-expression”; the turquoise line “interactions from curated databases”; the pink line “interactions experimentally determined”; the blue line “gene co-occurrence”; and the violet line “protein homology”.

## 3. Discussion

Dopamine, a neurotransmitter crucial for mood, pleasure, and reward regulation, has been suggested in certain studies to potentially contribute to breast cancer through dysregulated signaling pathways. This dysregulation could involve changes in dopamine receptors or levels within breast tissue, potentially impacting cell proliferation or apoptosis, both of which are processes associated with cancer development [34,35,36].

The notion that dopamine receptors might be influential in tumor progression gained initial support when it was observed that cancer patients undergoing antineoplastic treatment alongside antipsychotic drugs exhibited improved clinical responses [37,38].

In our study, we focused on genes selected in the microarray analysis whose fold change in the examined sections was at least 3-fold compared to control sections and that differentiated breast cancer sections regardless of the subtype compared to controls. Therefore, we focused on discussing changes in the expression of *DRD2*, *DRD3*, *DRD5*, *TGF-β-2*, and *CAV2* and the involvement of miRNAs in the control of their expression. We observed the overexpression of *DRD2* and *DRD3* and silencing of *DRD5*, *TGF-β-2*, and *CAV2* expression in tumor tissue compared to control sections. Pornour et al. assessed changes in the mRNA expression pattern of five dopamine receptors by qRT-PCR in peripheral blood mononuclear cells of Iranian women with breast cancer and in cell culture. Like us, these authors reported an increase in *DRD2* and *DRD3* expression and DRD5 silencing in women with breast cancer [39]. Hence, it is crucial to evaluate changes in these receptors as potential stressors before choosing suitable medications such as D2-like agonists for breast cancer treatment, following complementary testing. In turn, Prabju et al. [40] found that in glioblastoma multiforme, reduced DRD5 expression correlates with better clinical outcomes. These outcomes indicate that DRD5 acts as a negative modulator of DRD2 signaling and the tumor’s responsiveness to ONC201 DRD2 antagonism [40].

Regarding DRD3, its role in tumor biology remains poorly understood. Williford et al. observed that the expression of D3 receptors is elevated in glioblastoma, suggesting that therapy involving its antagonists could hold promise [41]. In turn, the expression profile at the mRNA and protein level of DRD5 was decreased in tumor samples in comparison to control samples. Leng et al. discovered that DRD5 was present in various human cancer cell types, including glioblastomas, colon cancer, and gastric cancer. Activation of DRD5 in these cell lines led to growth suppression, inhibition of MTOR activity, and induction of autophagy. This suggests a potential application of DRD5 agonists as a new therapeutic strategy for treating various human tumors and cancers [42]. In turn, Bai et al. suggested that the mechanism involved in the downregulation of DRD5 expression may be hypermethylation of the promoter region of the gene encoding DRD5 [43]. This is consistent with our observations, where we observed that although there is a potential interaction between DRD5 and mIR-4441, the expression of which is significantly lower in breast cancer, hypermethylation of the promoter region of the DRD5 gene is the primary mechanism that reduces its expression in breast cancer tissue.

The concurrent upregulation of both stimulatory (D1-like receptors) and inhibitory (D2-like receptors) dopamine receptors presents a formidable challenge in deciphering the role of dopaminergic signaling in breast cancer pathogenesis and progression. This phenomenon suggests that dysregulated dopaminergic signaling in breast cancer involves intricate and multifaceted mechanisms. It implies that distinct subtypes of breast cancer cells may exhibit disparate responses to dopamine, yielding heterogeneous outcomes on tumor proliferation, invasiveness, and metastasis. Moreover, the interplay between dopamine receptors and other signaling cascades, such as estrogen receptor signaling or growth factor pathways, likely further modulates cellular responses to dopamine stimulation. From a therapeutic perspective, the coexistence of dopamine receptors with opposing functions raises pertinent questions regarding the efficacy and safety of targeting dopaminergic signaling pathways for breast cancer treatment. While targeting a specific dopamine receptor subtype may attenuate tumor growth, it could inadvertently promote the proliferation of tumors expressing the antagonistic receptor subtype. Hence, a nuanced understanding of the distinct molecular profiles and signaling pathways within individual breast cancer subtypes is imperative when formulating tailored therapeutic strategies aimed at selectively modulating dopaminergic signaling. Additionally, probing the interplay between dopamine receptors and other signaling pathways, such as the MAPK/ERK or PI3K/AKT pathways, holds promise for elucidating the underlying mechanisms governing the diverse effects of dopaminergic signaling in breast cancer.

The changes in DRD3 and DRD5 expression we observed at the mRNA and protein levels may result, at least in part, from the regulatory role of miR-4441 and miR-16-5p. Reduced expression of miR-16-5p is characteristic of breast cancer, and in the case of the TNBC subtype, monitoring the expression of this miRNA may be a useful diagnostic and predictive marker [44]. In addition, it was determined that miR-16-5p shows reduced expression in breast cancer tissues. Furthermore, high expression of miR-16-5p appears to inhibit breast cancer occurrence by regulating AKT3 and suppressing the NF-κB pathway [45].

The TGF-β signaling pathway is noted for its involvement in a broad spectrum of cellular processes, often exerting opposing effects such as tumor suppression and tumor progression.

Chen et al. also observed significantly lower *TGF-β-2* mRNA expression in tumor tissue compared to control sections [46]. In addition, Gobbi et al. found that *TGF-β-2* mRNA transcriptional activity was downregulated in almost two-thirds of breast tumors, as was the expression of TGF-β receptor II (TGF-βRII) [47].

Literature data indicate a complex interaction between miRNAs and TGF-β signaling pathways involving various pairs such as miR-34 and miR-203 with SNAIL1, miR-200 and miR-205 with ZEB, and miR-1 and miR-200 with SLUG. Additionally, autocrine TGF-β/miR-200 negative feedback loops have been identified as central components of TGF-β induced epithelial–mesenchymal transition (EMT) [48,49,50].

In our analysis, we found that miR-141-3p is involved in regulating the expression of both TGF-β-2 and DRD2, exerting opposing effects visible in the concentration of proteins encoded by the mentioned genes. At the protein level, the concentration of TGF-β-2 was reduced in tumor sections compared to controls, while the DRD2 concentration was significantly higher in tumor sections, indicating that miRNA molecules not only act as a negative regulator at the post-transcriptional level [51,52,53]. Moreover, it should be remembered that the expression of miR-141-3p in breast cancer reported by other researchers was different. Zhang et al. and Song et al. reported a downregulation of the miRNA in question, as did we, while Dong et al. observed an increase in miR-141-3p expression in breast cancer [54,55,56].

The last transcript common to all five breast cancer types was *CAV2* mRNA, which was not regulated by miRNA. At both the mRNA and protein levels, we noted a reduction in CAV2 expression at the mRNA and protein levels.

In the past few years, accumulating evidence has indicated that CAV1 and CAV2 might possess oncogenic properties in breast cancer. Despite debates over the distribution of CAV1 and CAV2 in normal versus invasive breast cancer, recent research has reaffirmed their predominant expression in normal myoepithelial cells [57]. In addition, CAV2 expression may be a marker of treatment response in breast cancer, although further studies are needed [57,58]. Studies have indicated that its overexpression plays a crucial role in suppressing cancer cell migration and metastasis [59]. A prior investigation conducted by our team revealed that the depletion of cavin-2 can trigger epithelial–mesenchymal transition in breast cancer cells through the activation of the TGF-β signaling pathway [60]. A thorough analysis of cavin expression and function has provided evidence of the significant roles played by CAVIN1 and CAVIN2 in inhibiting the development of breast cancer. Both CAVIN1 and CAVIN2 showed notable downregulation in breast cancer tissues and were linked to patient prognosis. Among all caveolae-related genes, the downregulation of CAVIN2 in breast cancer tissues emerged as the most significant, independently assessing the predictive values in patients with breast cancer regardless of other CAVINs [60,61].

The current investigation presents several noteworthy limitations. Firstly, the sample size, particularly concerning subtypes such as HER2-positive and TNBC, may limit the generalizability of our findings, necessitating a larger cohort to enhance statistical robustness. Secondly, the study’s confinement to the Polish female population restricts the diversity of our patient cohort, potentially impacting the broader applicability of our results. Thirdly, while microarray analysis and qRT-PCR were instrumental in assessing gene expression patterns, their intrinsic limitations in capturing the nuanced intricacies of gene regulation prompt the consideration of alternative omics methodologies, such as RNA sequencing, to afford a more comprehensive understanding.

Nevertheless, it is pivotal to underscore the study’s commendable strengths. The adoption of a multifaceted approach, encompassing gene expression profiling, miRNA analysis, and protein quantification via ELISA, facilitated an exhaustive examination of dopamine-related pathways in breast cancer. The incorporation of diverse breast cancer subtypes amplifies the relevance and transferability of our findings across heterogeneous patient demographics. Furthermore, rigorous adherence to standardized protocols for gene expression and miRNA analysis ensured the fidelity and replicability of our outcomes. Leveraging reputable databases for miRNA target prediction augmented the elucidation of potential regulatory mechanisms underlying dopamine-mediated gene expression alterations.

In light of these strengths and limitations, future research avenues should be pursued. Conducting longitudinal studies to monitor temporal changes in dopaminergic signaling pathways in breast cancer patients could unveil dynamic insights into the progression and treatment response of the disease. Elucidating the specific molecular pathways and cellular processes governed by dopaminergic abnormalities through mechanistic studies employing in vitro and in vivo models holds promise for deciphering the underlying pathophysiology. Integration of omics approaches, encompassing gene expression profiling alongside proteomics, metabolomics, or epigenomics analyses, could provide a holistic understanding of the intricate regulatory networks implicated in breast cancer. This integrative approach may unearth novel biomarkers, therapeutic targets, and predictive signatures conducive to personalized medicine strategies. Additionally, translating research findings into clinical practice by validating the biomarkers or therapeutic targets identified in preclinical studies via clinical trials or retrospective analyses of patient cohorts holds the potential for advancing diagnostic precision and therapeutic efficacy tailored to the molecular intricacies of breast cancer subtypes.

## 4. Materials and Methods

### 4.1. Subjects

The study encompassed patients diagnosed with five distinct breast cancer subtypes, namely luminal A (*n* = 130), luminal B (*n* = 196, comprising HER2-negative (*n* = 100) and HER2-positive (*n* = 96) subgroups), HER2-positive (*n* = 36), and triple-negative breast cancer (TNBC) (*n* = 43). These patients underwent surgical procedures during which tumor tissue was excised alongside a margin of healthy tissue as a control (Table 4).

Detailed characteristics of patients belonging to each of the five subtypes are provided in Table 4. Notably, all patients were classified according to the Tumor, Nodules, and Metastases (TNM) classification system as T1N0M0 [62].

During each operation, the pathomorphological team assessed whether the tumor lesion was removed with a margin of healthy tissue (immunohistochemistry staining). When this margin was not obtained in the intraoperative assessment, the scope of surgery was expanded. Thus, on the basis of the pathomorphological assessment performed during surgery, a distinction was made between tumor-affected tissue (study) and non-tumor-affected tissue (control).

### 4.2. Isolation of Total Ribonucleic Acid (RNA)

The process of extracting total RNA from tissues commenced with the utilization of TRIzol reagent (Invitrogen Life Technologies, Carlsbad, CA, USA; Catalog number: 15596026), meticulously following the manufacturer’s protocol. Subsequently, the RNeasy mini kit (QIAGEN, Hilden, Germany; Catalog number: 74104) was employed to purify the isolated RNA, ensuring the removal of impurities and contaminants. To further refine the RNA samples, treatment with DNase I (Fermentas International Inc., Burlington, ON, Canada; Catalog number: 18047019) was performed to eliminate any potential genomic DNA contamination.

Qualitative evaluation of the extracted RNA was conducted through 1% agarose gel electrophoresis supplemented with 0.5 mg/mL ethidium bromide, facilitating a visualization and assessment of RNA integrity. Additionally, a quantitative assessment of the RNA concentration was carried out by measuring the absorbance at 260 nm, providing insight into the yield and purity of the RNA samples obtained.

### 4.3. Microarray Profiling of Dopamine-Related Genes

The mRNA names and their ID number were determined from the Affymetrix NetAffx™ Analysis Center database after entering the phrase “dopamine” (http://www.affymetrix.com/analysis/index.affx) (accessed on 1 August 2021). The analysis showed that 175 mRNAs were related to dopamine signaling paths.

The comparative expression analysis of dopamine-related genes in tumor tissues as opposed to control tissues was conducted utilizing the HG-U 133_A2 microarray platform (Affymetrix, Santa Clara, CA, USA) and the GeneChip™ 3′ IVT PLUS reagent kit (Affymetrix; Catalog Number 902416), adhering strictly to the manufacturer’s protocols and methodologies outlined in prior research endeavors. Among the 22,277 mRNA probes present on the microarray plate, a total of 65 probes were specifically linked to the histaminergic system, as identified through the Affymetrix NetAffx Analysis Center database. The microarray analysis protocol entailed the initial synthesis of double-stranded complementary DNA (cDNA) utilizing the GeneChip 30IVT Express kit (Thermo Fisher Scientific, Waltham, MA, USA), followed by subsequent steps of RNA amplification and fragmentation. Following these preparatory steps, the resulting amplified RNAs (aRNAs) were subjected to hybridization, and the resultant fluorescence intensity was quantified utilizing an Affymetrix Gene Array Scanner 3000 7G, coupled with the Gene Chip^®^ Command Console^®^ Software 6.0+ (Affymetrix, Santa Clara, CA, USA). This comprehensive approach facilitated the thorough examination of differential gene expression patterns within the histaminergic system, shedding light on potential molecular alterations associated with tumorigenesis.

### 4.4. Comprehensive Microarray Profiling of Dopamine-Related miRNAs and Their Potential Impact on Gene Expression

To delve into the intricate role of dopamine-related miRNAs and their potential influence on the expression of analyzed genes, we conducted a microarray analysis utilizing the GeneChip miRNA 2.0 Array (Affymetrix), a widely recognized commercial platform renowned for its reliability and precision. The microarray profiling procedure strictly adhered to the manufacturer’s instructions, ensuring standardized and reproducible results.

Identification of differentially expressed miRNAs between tumor and control tissues, crucial for modulating the expression of differentially expressed mRNAs, was meticulously carried out using two reputable databases: TargetScan (http://www.targetscan.org/) (accessed on 1 August 2021) [63] and miRanda (http://mirdb.org) (accessed on 1 August 2021) [64]. These databases provided invaluable insights into potential miRNA–mRNA interactions, aiding in the elucidation of underlying regulatory mechanisms [64,65].

In our analysis, a predicted target with a prediction score surpassing 80 was considered highly credible, indicative of a robust miRNA–mRNA interaction. However, it is essential to exercise caution when interpreting results with prediction scores below 60, as these may require additional corroborating evidence to validate their authenticity [64,65]. By integrating data from multiple sources and employing stringent criteria for target prediction, we aimed to ensure the reliability and accuracy of our findings, paving the way for a comprehensive understanding of the intricate regulatory networks governing dopamine-related miRNA-mediated gene expression alterations.

### 4.5. Quantitative Reverse Transcription Polymerase Chain Reaction (qRT-PCR) Analysis

To validate the microarray data, qRT-PCR was conducted for selected genes. The SensiFast SYBR No-ROX One-Step kit (Bioline, London, UK) was employed in accordance with the manufacturer’s guidelines. Expression profiles of specific genes were presented using the 2^−ΔΔCt^ method, where a fold change equal to 1 represented the control, greater than 1 indicated overexpression, and less than 1 indicated silencing. β-actin (*ACTB*) was utilized as an internal control for normalization. Detailed primer sequences are provided in Table 5 for reference.

### 4.6. Enzyme-Linked Immunosorbent Assay (ELISA)

This study’s concluding phase assessed protein concentrations. To accurately quantify protein concentrations, we used specific ELISA kits in accordance with the manufacturer’s instructions: Human Dopamine Receptor D5 Kit (MyBioSource, Inc., San Diego, CA, USA, Cat No. MBS724527), Human Dopamine Receptor D2 Kit (MyBioSource, Inc., San Diego, CA, USA, Cat No. MBS723432), Human Dopamine Receptor D3 Kit (MyBioSource, Inc., San Diego, CA, USA, Cat No. MBS722010), Human Caveolin 2 kit (MyBioSource, Inc., San Diego, CA, USA, Cat No. MBS1607726) and Human TGF beta 2 Kit (MyBioSource, Inc., San Diego, CA, USA, Cat No. MBS824902).

### 4.7. Statistical Analysis

Statistical analyses were conducted utilizing licensed versions of Statistica 13.0 PL (StatSoft, Cracow, Poland) and the Transcriptome Analysis Console programs (Affymetrix). To assess the normality of data distribution, the Shapiro–Wilk test was employed, with significance set at *p* < 0.05. Mean differences were analyzed using analysis of variance (ANOVA) with the Benjamin–Hochberg correction, followed by Tukey’s post hoc test (*p* < 0.05) or Student’s *t* test, depending on the specific comparison. The Kaplan–Meier plotter (http://kmplot.com/; accessed on 5 May 2024) was used to plot the overall survival status in every group [66,67].

The relationships between genes were thoroughly examined using the Search Tool for the Retrieval of Interacting Genes/Proteins (STRING Database 11.0; accessed 5 May 2024). Within the STRING database, the parameter strength Log10 (observed/expected) quantifies the extent of the enrichment effect. It reflects the ratio of (1) the number of proteins annotated with a specific term within the network to (2) the expected number of proteins annotated with that term in a randomly generated network of equivalent size. Conversely, the false discovery rate parameter gauges the significance of the enrichment. Reported are *p*-values adjusted for multiple testing within each category employing the Benjamini–Hochberg procedure [68].

#### Sample Size Analysis

Sample size calculations were made using a sampling calculator [69]. Assuming a confidence interval value of 95% and a total of about 19,620 women diagnosed with breast cancer in Poland in 2019 [70], the recommended number of participants for the study was 377.

Furthermore, according to the literature data [71,72], luminal A breast cancer accounts for 23.7% of all breast cancer subtypes (in our study 32.10%); luminal B HER2− subtype accounts for 38.8% of all breast cancer subtypes (in our study 38.8%); luminal B HER2+ subtype accounts for 14% of all breast cancer subtypes (23.7% in our study); HER2+ subtype accounts for 11.2% of all breast cancer subtypes (8.89% in our study); and TNBC subtype accounts for 12.3% of all breast cancer subtypes (10.62% in our study).

## 5. Conclusions

Our analysis suggests the potential involvement of the dopaminergic system in breast cancer; however, further research is warranted to confirm its importance. The observed expression profiles of mRNAs and miRNAs indicate that they possibly serve as molecular markers, although additional studies are necessary to validate their utility. We noted the overexpression of DRD2 and DRD3, along with the concurrent silencing of DRD5 expression, which may suggest the presence of dopaminergic alterations in breast cancer patients. This observation may be influenced by the activity of miR-141-3P, miR-16-5p, and miR-4441, which might regulate processes such as proliferation or metastasis. Nevertheless, caution should be exercised in interpreting these findings, as further investigations are required to elucidate the precise role of dopaminergic abnormalities in breast cancer pathogenesis.

## Figures and Tables

**Table 1:** Changes in the expression profile of genes differentiating tumor samples compared to control tissues (−3.0 < FC > 3.0; *p* < 0.05).

ID	mRNA	Luminal A vs. Control	Luminal B HER2− vs. Control	Luminal B HER2+ vs. Control	Non-Luminal HER2+ vs. Control	TNBC vs. Control
214652_at	*DRD1*	6.78 ± 0.18	3.45 ± 0.12	3.54 ± 0.88	3.98 ± 0.12	7.87 ± 1.09
216924_s_at	*DRD2*	14.56 ± 0.91	14.78 ± 0.91	15.98 ± 1.34	11.99 ± 2.01	10.98 ± 0.91
216938_x_at		14.65 ± 2.11	15.01 ± 0.91	14.98 ± 1.45	10.98 ± 1.98	10.54 ± 1.76
206590_x_at		14.43 ± 1.98	14.56 ± 0.76	15.56 ± 1.67	11.65 ± 2.11	10.19 ± 2.10
211625_s_at	*DRD3*	6.98 ± 0.87	7.51 ± 0.16	4.56 ± 0.43	5.67 ± 0.56	9.87 ± 0.42
214559_at		4.56 ± 0.78	5.67 ± 0.19	7.12 ± 0.54	3.09 ± 0.13	5.67 ± 0.56
208486_at	*DRD5*	−4.57 ± 0.91	−11.98 ± 1.23	−6.98 ± 1.87	−13.01 ± 0.81	−4.56 ± 0.19
209908_s_at	*TGF-β-2*	−5.66 ± 0.81	−12.09 ± 0.18	−11.09 ± 0.23	−8.78 ± 0.76	−9.98 ± 0.98
209909_s_at		−5.45 ± 0.44	−12.76 ± 0.87	−10.98 ± 0.65	−8.99 ± 0.34	−8.98 ± 0.65
220406_at		−5.69 ± 0.23	−11.23 ± 0.81	−10.11 ± 0.41	−9.09 ± 0.65	−9.99 ± 0.17
220407_s_at		−5.98 ± 0.87	−12.98 ± 0.14	−11.34 ± 0.56	−10.34 ± 0.98	−10.09 ± 0.19
203323_at	*CAV2*	−3.45 ± 0.23	−4.56 ± 0.65	−4.09 ± 0.34	−3.41 ± 0.76	−5.67 ± 0.18
203324_s_at		−3.78 ± 0.54	−4.09 ± 0.76	−4.15 ± 0.77	−3.98 ± 0.11	−5.19 ± 0.19
213426_s_at		−3.87 ± 0.45	−4.13 ± 0.18	−4.78 ± 0.66	−4.01 ± 0.19	−5.13 ± 0.54
207201_s_at	*SLC22A1*	3.98 ± 0.12	4.01 ± 0.14	3.78 ± 0.19	5.87 ± 0.24	5.11 ± 0.98
209687_at	*CXCL12*	8.98 ± 1.01	6.19 ± 0.98	3.01 ± 0.24	5.01 ± 0.23	6.17 ± 0.51
203666_at		8.17 ± 0.87	6.22 ± 0.87	4.56 ± 0.43	5.07 ± 0.67	6.23 ± 0.78
204621_s_at	*NR4A2*	4.56 ± 0.18	4.71 ± 0.18	4.99 ± 0.91	4.76 ± 0.34	7.09 ± 0.32
204622_x_at		4.16 ± 0.71	4.71 ± 0.23	4.89 ± 0.12	5.09 ± 0.67	7.12 ± 0.14
216248_s_at		4.76 ± 0.56	4.55 ± 0.44	4.51 ± 0.54	5.19 ± 0.13	7.56 ± 0.71
220805_at	*HRH2*	4.15 ± 0.23	4.76 ± 0.24	4.15 ± 0.81	5.12 ± 0.45	7.18 ± 0.65
209569_x_at	*NSG1*	3.45 ± 0.19	3.09 ± 0.18	3.18 ± 0.34	3.98 ± 0.19	3.91 ± 0.13
209570_s_at		3.21 ± 0.22	3.12 ± 0.14	3.19 ± 0.33	3.31 ± 0.22	3.78 ± 0.17
213533_at		3.56 ± 0.24	3.14 ± 0.23	3.29 ± 0.31	3.11 ± 0.21	3.81 ± 0.42

*DRD2*, dopamine receptor 2; *DRD3*, dopamine receptor 3; *DRD5*, dopamine receptor 5; *TGF-β-2*, transforming growth factor beta 2; *CAV2*, caveolin 2; *SLC22A1*, solute carrier family 22 (organic cation transporter), member 1; *CXCL1*2, chemokine (C-X-C motif) ligand 12; *DRD1*, dopamine receptor 1; *NR4A2*, nuclear receptor subfamily 4, group A, member 2; *HRH2*, histamine receptor H2; *NSG1*, neuron-specific gene family member 1.

**Table 2:** The expression profile of miRNAs potentially regulated by selected mRNAs in different breast cancer subtype tissues in comparison to control tissues.

mRNA	miRNA	Target Score	Luminal A vs. Control (FC)	Luminal B HER2− vs. Control (FC)	Luminal B HER2+ vs. Control (FC)	Non-Luminal Her2+ vs. Control (FC)	TNBC vs. Control (FC)
*DRD2* *TGF-β-2*	hsa-miR-141-3p	94 95	−3.98 ± 0.19	−3.45 ± 0.12	−3.12 ± 0.23	−7.87 ± 0.18	−3.01 ± 0.32
*DRD3* *DRD5*	hsa-miR-4441	90 93	−6.13 ± 0.19	−4.56 ± 0.76	−5.66 ± 0.21	−3.45 ± 0.18	−3.98 ± 0.87
*DRD5*	hsa-miR-16-5p	89	−4.10 ± 0.98	−4.87 ± 0.19	−4.12 ± 0.98	−3.65 ± 0.98	−5.03 ± 0.81

*DRD2*, dopamine receptor 2; *DRD3*, dopamine receptor 3; *DRD5*, dopamine receptor 5; *TGF-β-2*, transforming growth factor beta 2; FC, fold change. Data are presented as mean ± standard deviation.

**Table 3:** The concentration of selected proteins related to the dopaminergic system in different subtypes of breast cancer and control tissue.

Protein	Control Tissue	Luminal A	Luminal B HER2−	Luminal B HER2+	Non-Luminal HER2+	TNBC
DRD2 [ng/mL]	3.13 ± 0.98	10.91 ± 0.98 *	6.87 ± 1.12 *	6.17 ± 1.91 *	9.99 ± 1.67 *	10.98 ± 2.12 *
DRD3 [ng/mL]	6.71 ± 4.56	10.98 ± 2.10 *	9.10 ± 0.98 *	12.87 ± 2.45 *	11.88 ± 2.65 *	13.12 ± 1.45 *
DRD5 [ng/mL]	22.98 ± 5.06	16.81 ± 2.18 *	18.81 ± 2.34 *	16.87 ± 2.19 *	17.09 ± 0.51 *	5.98 ± 0.43 *
TGF-β-2 [pg/mL]	4.15 ± 2.18	1.09 ± 0.13 *	below detection threshold *	below detection threshold *	below detection threshold *	below detection threshold *
CAV2 [ng/mL]	2.34 ± 0.19	0.76 ± 0.18 *	0.65 ± 0.04 *	0.98 ± 0.09 *	0.34 ± 0.02 *	0.14 ± 0.06 *

DRD2, dopamine receptor 2; DRD3, dopamine receptor 3; DRD5, dopamine receptor 5; TGF-β-2, transforming growth factor beta 2; CAV2, caveolin 2. Data are presented as mean ± standard deviation; * *p* < 0.05 vs. control tissue.

**Table 4:** Patient characteristics.

Molecular Type	Degree of Histological Malignancy			Age		BMI [kg/m^2^]
	G1	G2	G3	<50 Years	>50 Years	
Luminal A	23 (18%)	48 (37%)	59 (45%)	43 (33%)	87 (67%)	30.78 ± 2.76
Luminal B HER2−	31 (31%)	57 (57%)	12 (12%)	32 (32%)	68 (68%)	30.18 ± 4.56
Luminal B HER+	23 (24%)	57 (59%)	16 (17%)	19 (20%)	77 (80%)	32.09 ± 6.19
Non-luminal HER2+	9 (25%)	12 (33%)	15 (42%)	9 (25%)	27 (75%)	33.18 ± 5.67
TNBC	14 (32%)	21 (49%)	8 (19%)	10 (23%)	33 (77%)	34.67 ± 2.98

Data are presented as number of cases and (percentage) or mean ± standard deviation; HER, human epidermal growth factor receptor 2; TNBC, triple-negative breast cancer; BMI, body mass index.

**Table 5:** The sequence of primers used in qRT-PCR.

mRNA	Nucleotide Sequence	
*DRD2*	Forward	5′-GATTTGGAGAGGTAGAATTGGAGT-3′
	Reverse	5′-CAACCCAAAACATAACCAATATAAC-3′
*DRD3*	Forward	5′-GGTTTTTATTGTTTGTTGGTTGTTT-3′
	Reverse	5′-ATATTCCCTCTTCTACTCCCTCAAC-3′
*DRD5*	Forward	5′-TAGTTTAATTGGTATAGGGATTAGG-3′
	Reverse	5′-TAAAATCACAATTCTCTACATTCAC-3′
*TGF-β-2*	Forward	5′-TACTACGCCAAGGAGGTTTACAAA-3 ′
	Reverse	5′-TTGTTCAGGCACTCTGGCTTT-3 ′
*CAV2*	Forward	5′-TGTTTTTGGTTATTTTTTTGGTTTT-3′
	Reverse	5′-TCCAAATATTCAATCCTAACTCAATTAC-3′
*ACTB*	Forward	5′-TCACCCACACTGTGCC CATCTACGA-3′
	Reverse	5′-CAGCGGAACCGCTCATTGCCAATGG-3′

*DRD2*, dopamine receptor 2; *DRD3*, dopamine receptor 3; *DRD5*, dopamine receptor 5; *TGF-β-2*, transforming growth factor beta 2; *CAV2*, caveolin 2; ACTB, β-actin.

## Data Availability

The data used to support this study’s findings are included in this article. The data will not be shared due to third-party rights and commercial confidentiality.
